# Association of Electrocardiogram Abnormalities with Clinical Outcomes in Emergency Department Sepsis Patients

**DOI:** 10.5811/westjem.50775

**Published:** 2026-02-11

**Authors:** Praew Kotruchin, Mingkamon Chuehongthong, Thanat Tangpaisarn, Nattapat Serewiwattana, Pariwat Phungoen, Thapanawong Mitsungnern, Marturod Buranasakda

**Affiliations:** * Khon Kaen University, Faculty of Medicine, Department of Emergency Medicine, Khon Kaen, Thailand; † Khon Kaen University, Faculty of Medicine, Queen Sirikit Heart Center of the Northeast, Khon Kaen, Thailand

## Abstract

**Introduction:**

Sepsis, a critical condition caused by dysregulated host responses to infection, frequently involves cardiac complications. Electrocardiogram (ECG) provides valuable insights into the cardiovascular status of sepsis patients and may guide early interventions. However, comprehensive data on ECG patterns in sepsis patients within the emergency department (ED) is limited. In this study we aimed to identify common ECG rhythms and patterns in sepsis patients presenting to the ED and analyze their association with poor clinical outcomes, including intensive care unit (ICU) admission, prolonged hospital stay (> 14 days), and in-hospital mortality.

**Methods:**

We conducted a retrospective observational study using data from 3,598 adult sepsis patients presenting to the ED of Srinagarind Hospital, Khon Kaen, Thailand, between January–December 2023. ECG abnormalities were extracted from the automated ECG interpretation system. Cardiologists reviewed only ECGs flagged as potential acute infarction or ST elevation to confirm acute coronary syndrome patterns. We analyzed associations between ECG abnormalities and clinical outcomes using univariate logistic regression models.

**Results:**

Common ECG rhythms in sepsis patients included sinus rhythm (41.7%), sinus tachycardia (39.0%), and atrial fibrillation/flutter (8.8%). The automated algorithm identified prolonged QT intervals (54.4%) and ST elevation in 10.4% of patients; however, only 1.7% met cardiologist-confirmed criteria for acute coronary syndrome. Compared with patients with better outcomes, those with poor outcomes more frequently had atrial fibrillation/flutter (14.9 vs. 7.5%), new-onset atrial fibrillation/flutter (6.0 vs. 2.8%), QT prolongation (61.6 vs. 52.9%), and abnormal T waves (10.9 vs. 8.4%), corresponding to odds ratios of 2.19 (95% CI, 1.77–2.69), 2.24 (1.50–3.28), 1.43 (1.20–1.70), and 1.34 (1.01–1.76), respectively.

**Conclusion:**

Certain ECG abnormalities in sepsis patients are associated with adverse clinical outcomes. Incorporating ECG assessments into sepsis protocols may enhance the early identification of high-risk patients and improve management strategies in the ED.

## INTRODUCTION

Sepsis is a life-threatening organ dysfunction caused by a dysregulated host response to infection and remains a significant cause of morbidity and mortality worldwide.^1^ Rapid identification and management of sepsis are critical to improve patient outcomes. Among the many organs affected by sepsis, the heart is one of the most frequently impacted.^2^ Cardiac complications are common in sepsis and can range from transient arrhythmias to severe myocardial dysfunction.^2^–^6^ Lin et al conducted a meta-analysis that identified sepsis-induced cardiomyopathy as a condition associated with increased one-month mortality, highlighting the importance of addressing cardiac dysfunction in sepsis patients.^7^ Understanding common electrocardiogram (ECG) patterns in sepsis patients can aid clinicians in detecting and promptly managing cardiac complications. This can enhance risk stratification, inform therapeutic decisions, and potentially reduce the incidence of adverse cardiac events and mortality.^8^ICU or can be discharged. Current risk stratification tools are based on measurements of vital parameters at a single timepoint. Here, we performed a time, frequency, and trend analysis on continuous electrocardiograms (ECG

Electrocardiography is a non-invasive, readily available diagnostic tool that can provide valuable insights into the cardiovascular status of sepsis patients upon presentation to the emergency department (ED). Early recognition of cardiac complications through ECG can guide treatment and improve prognosis. Previous studies have highlighted various ECG abnormalities in sepsis patients, including both rhythm and pattern components, such as sinus tachycardia, supraventricular tachyarrhythmias (eg, atrial fibrillation), abnormal QRS complexes (eg, decreased QRS amplitudes, increased QRS duration), ST-T changes (eg, ST elevation, ST depression, T-wave inversion), prolonged QT intervals, and other patterns such as bundle branch block and Brugada pattern.^2^–^4^,^6^,^9^–^15^ Despite these findings, comprehensive data on the prevalence and specific types of ECG patterns observed in sepsis patients during ED presentation are limited.

In this study we aimed to identify and characterize the common ECG rhythms and patterns observed in sepsis patients presenting to the ED. By analyzing the ECG data of these patients, we aimed to clarify the correlation between specific ECG findings and poor clinical outcomes, including the need for intensive care unit (ICU) admission, prolonged hospital stay (> 14 days), and in-hospital mortality.

## METHODS

### Study Design and Ethical Approval

We conducted this retrospective study at the ED of Srinagarind Hospital, a tertiary-care facility affiliated with Khon Kaen University, and followed the Strengthening the Reporting of Observational Studies in Epidemiology guidelines for observational cohort studies. We focused on sepsis patients who presented in the ED between January 1–December 31, 2023. The study received approval from the Human Research Ethics Committee of Khon Kaen University (HE671521). Informed consent from patients was waived due to the retrospective nature of the data collection, which involved pre-existing data.

### Study Population

Adult patients ≥ 18 years of age who presented to the ED with sepsis were included. Sepsis was defined as having a suspected or confirmed infection and meeting at least two criteria from either the Systemic Inflammatory Response Syndrome (SIRS) or the Quick Sequential Organ Failure Assessment (qSOFA).^1^,^16^ We identified sepsis cases via structured clinical flags, SIRS-based vital sign triggers, and qSOFA parameters from triage documentation. Patients were excluded if they had incomplete or missing ECG or clinical data. It is important to note that ECGs were not routinely performed for all sepsis patients in the ED, as their acquisition was based on clinical suspicion of cardiac involvement rather than a standardized protocol.

**Population Health Research Capsule**

What do we already know about this issue?

*Sepsis commonly affects cardiac function, and electrocardiogram (ECG) abnormalities may signal worse outcomes*.

What was the research question?

*Which ECG abnormalities in ED sepsis patients are associated with poor clinical outcomes?*

What was the major finding of the study?

*Atrial fibrillation/flutter (OR 2.19, < .001) and QT prolongation (OR 1.43, < .001) were linked to poor outcomes*.

How does this improve population health?

*Identifying high-risk sepsis patients early using ECG findings may help guide timely interventions and improve outcomes in resource-limited ED settings*.

### Data Collection

We conducted the data collection process as follows:

1. Pre-defined dataset: A specific dataset was established before extraction, which included the following categories:
  - Demographics: sex, age, and underlying comorbidities.
  - Vital signs: Body temperature (BT), heart rate (HR), respiratory rate (RR), systolic and diastolic blood pressure (SBP and DBP), and peripheral oxygen saturation (SpO_2_).
  - Laboratory results: white blood cell count (WBC), serum creatinine, potassium, calcium, magnesium, bicarbonate, and lactate levels.
  - ECG findings: Rhythm classification (eg, sinus rhythm, atrial fibrillation/flutter, sinus tachycardia) and specific ECG patterns (eg, QT prolongation, ST-T abnormalities, T-wave abnormalities). Findings were classified into two predefined categories: 1) rhythm abnormalities, including sinus rhythm, sinus tachycardia, and atrial fibrillation/flutter; and 2) specific ECG pattern abnormalities, including QTc prolongation, ST-T abnormalities, and T-wave abnormalities.
2. Data extraction: Trained data collectors retrieved the specified data set from Srinagarind Hospital’s electronic health record (EHR) database. The ECG data were obtained from the automated ECG interpretations generated by the Philips PageWriter TC20 12-lead ECG machine, which uses the Philips DXL ECG Algorithm (Philips Healthcare, Andover, Massachusetts, USA). QTc intervals were calculated automatically by the Philips DXL system using the Bazett correction formula. No manual QTc rcalculation was performed.
3. Data validation: The extracted data were manually reviewed for validity and accuracy; missing ECG or laboratory data resulted in case exclusion; no imputation was performed; all variables were directly extracted from the EHR.
4. Categorization of ECG findings: Automated ECG interpretations were categorized to match the rhythm classifications and ECG patterns defined in Step 1.
5. Identification of new ECG abnormalities: ECGs requiring assessment for new-onset abnormalities, specifically atrial fibrillation/flutter and left bundle branch block were individually reviewed by a study physician. New-onset atrial fibrillation/flutter was defined as atrial fibrillation or flutter appearing on the first ECG obtained at our hospital without any previously documented arrhythmia in the EHR. Because outside-hospital ECGs were not consistently available, misclassification of pre-existing atrial fibrillation/flutter as new-onset is possible. New-onset atrial fibrillation/flutter was analyzed as a subgroup of the overall atrial fibrillation/flutter category. Similarly, a left bundle branch block identified on the first hospital ECG without prior documentation was classified as being new.
6. Review of acute infarction or ST elevation: Only ECGs flagged by the Philips DXL automated interpretation system as “acute infarction/ST elevation” were individually reviewed by a cardiologist. The cardiologist (PK) categorized these ECGs using standard acute coronary syndrome criteria. ECGs meeting ST-elevation myocardial infarction (STEMI) criteria were classified as STEMI, while ECGs showing ischemic changes without meeting STEMI thresholds (eg, dynamic ST-T abnormalities or new Q waves) were classified as non-ST elevation acute coronary syndrome.^17^ Thus, acute coronary syndrome in this study includes both STEMI and non-ST elevation acute coronary syndrome.
7. Retrospective chart review framework: This retrospective chart review followed key elements recommended by Worster et al for medical record review studies in emergency medicine research.^18^ Specifically:
  - We used a clearly defined research objective.
  - Inclusion and exclusion criteria were explicitly described in the Methods section.
  - A standardized and pre-specified data abstraction form was applied to all records.
  - Trained abstractors performed data collection, and data quality was monitored through manual validation.
  - ECGs requiring interpretation were reviewed by clinicians blinded to patient outcomes.

### Outcomes

The primary outcome of this study was to evaluate the association between abnormal ECG findings and poor clinical outcomes in adult patients with sepsis presenting to the ED. Poor clinical outcome was defined as a composite of at least one of the following: ICU admission, in-hospital death, or hospital stay >14 days.^19^–^21^ This composite endpoint was chosen to capture a broad range of clinically meaningful adverse outcomes that reflect significant resource use and morbidity, a common approach in emergency care and sepsis research for evaluating severe illness. While ICU admission and mortality represent established clinical endpoints, we included prolonged hospitalization as a proxy for severe illness or complications during admission. The secondary outcome was to describe and identify the most common ECG rhythms and patterns observed in sepsis patients presenting to the emergency department.

### Statistical Analysis

Patients were categorized into two groups based on clinical outcomes: poor outcome and better outcome. A poor outcome was defined as requiring admission to the ICU, a prolonged hospital stay (> 14 days), or in-hospital mortality. Patients who did not meet these criteria were classified as having a better outcome. Continuous variables were summarized as mean (standard deviation,), based on the central limit theorem, due to the large sample size, which allows the mean to be a reliable estimator regardless of the underlying data distribution. Categorical variables were presented as frequencies and percentages.

Inferential statistics were carried out using either the *t*-test or the Mann-Whitney U test for continuous variables and the chi-square test or Fisher exact test for categorical variables. Additionally, univariate logistic regression analysis was performed to examine the association between ECG patterns and poor clinical outcomes. A *P*-value of less than 0.05 was considered statistically significant. All statistical analyses were conducted using R Statistical Software v4.4.2 (R Foundation for Statistical Computing, Vienna, Austria).

## RESULTS

### Baseline Characteristics

During the study period, a total of 61,919 patients presented to the ED, of whom 8,805 were identified as having sepsis. After excluding 3,447 patients due to no ECG data and 1,760 patients due to missing clinical data, 3,598 patients remained eligible for analysis. (Figure 1) Among the 3,598 sepsis patients included in the final analysis, 3,472 met SIRS criteria, 737 met qSOFA criteria, and 611 met both.

The baseline characteristics of the study population are presented in Table 1. The mean age of patients was 63 years (SD 18), with 55% being male. Patients with poor outcomes were a higher proportion of male (59.9% vs.54.1%, *P* < .01) and exhibited higher respiratory rates (27 vs. 26 breaths/minute, *P* < 0.001), but lower systolic blood pressure (129 vs 135 millimeters mercury, *P* < 0.001). Peripheral oxygen saturation was also notably lower in the poor outcome group (94.2% vs. 95.6%, *P* = 0.007). Although the differences showed statistically significant differences between groups, the magnitude of difference was minimal and likely not clinically relevant.

Certain comorbidities were significantly associated with poor outcomes, including known atrial fibrillation/flutter (24.0% vs. 12.6%, *P* < .001), congestive heart failure (15.6% vs. 11.4%, *P* < .01), pulmonary embolism (6.8% vs. 4.0%, *P* = .002), liver disease (9.9% vs. 6.5%, *P* < .01). In terms of laboratory results, the poor outcome group had higher WBC counts (13,738 vs. 12,444 cells/mm^3^, *P* < 0.001), potassium levels (4.12 vs. 4.06 mEq/L, *P* = 0.04), and serum lactate levels (3.1 vs. 2.7 millimoles per liter, *P* < 0.001). Conversely, they had lower calcium levels (8.5 vs. 8.7 milligrams per deciliter, *P* < 0.001) and bicarbonate level (20.6 vs 20.9 milliequivalents/L, P < 0.04).

### Clinical Outcomes

Due to overlapping criteria, where some patients met multiple poor outcome definitions, the total number of unique patients classified as having poor outcomes was 649 (18.0%) of the study population. This included 142 patients (21.8%) who required ICU admission, 167 patients (25.7%) who experienced in-hospital mortality, and 426 patients (65.6%) who had prolonged hospital stays > 14 days.

### Electrocardiograph Rhythms in Sepsis Patients

The ECG rhythm patterns are summarized in Table 2. The most common observed rhythm was sinus rhythm, present in 42% of patients, followed by sinus tachycardia in 39% and atrial fibrillation/flutter in 8.8%. Patients with poor outcomes were significantly less likely to have sinus rhythm compared to those with better outcomes (34.5% vs. 43.3%, *P* < 0.001). Conversely, atrial fibrillation/flutter was more common among patients with poor outcomes (14.9% vs. 7.5%, *P* < 0.001). Further emphasizes these associations, showing that sinus rhythm was protective against poor outcomes (odds ratio (OR) 0.69, 95% CI, 0.58–0.82). In contrast, atrial fibrillation/flutter, especially when new-onset, was associated with adverse outcomes (OR 2.23, 95% CI, 1.50–3.28).

### Electrocardiograph Patterns in Sepsis Patients

The ECG patterns are detailed in Table 3. The most prevalent abnormality observed was QT prolongation, found in 54.4% of patients. ST elevation was flagged by the automated ECG interpretations algorithm in 10.4% of cases; however, after cardiologist review, only 1.7% met criteria for acute coronary syndrome. Abnormal T waves were observed in 8.9% of patients and included non-specific repolarization changes such as flattening, inversion, or biphasic T waves. Certain patterns showed a significant association with poorer outcomes, including QT prolongation (61.6% in patients with poor outcomes compared to 52.9% in those without, *P* < .001), abnormal T waves (10.9% vs. 8.4%, *P* = 0.04), and right bundle branch block (4.3 vs. 2.8%, *P* = 0.05). further highlights these associations, with QT prolongation increasing the odds of poor outcomes (OR 1.43, 95% CI: 1.20–1.70) and abnormal T waves showing a similar trend (OR 1.34, 95% CI: 1.01–1.76). Right bundle branch block also demonstrated a potential association with poor outcomes (OR 1.56, 95% CI: 0.99–2.38).

## DISCUSSION

Our study demonstrates the significant prevalence and prognostic value of ECG abnormalities in sepsis patients presenting to the ED. We emphasize the importance of ECG as a non-invasive diagnostic and prognostic tool that can aid in early risk stratification and management decisions. The most frequently observed rhythm was sinus rhythm, in 42% of patients. Sinus rhythm was significantly associated with better outcomes, emphasizing its role in maintaining cardiovascular stability during sepsis. Preserving sinus rhythm reflects a more stable autonomic and metabolic state, as sepsis-induced myocardial injury often disrupts normal conduction pathways.^8^ Recent studies have further supported this finding, with van Wijk et al demonstrating that heart rate variability analysis can identify early clinical deterioration in sepsis, indicating a potential for advanced risk stratification tools.^8^ ICU or can be discharged. Current risk stratification tools are based on measurements of vital parameters at a single timepoint. Here, we performed a time, frequency, and trend analysis on continuous electrocardiograms (ECG Sinus tachycardia, found in 39% of patients, is a common response to systemic hypoperfusion, fever, or inflammation in sepsis. While not directly correlated with poor outcomes in this study, its presence often signals compensatory mechanisms in the early stages of sepsis. Persistent or extreme tachycardia may exacerbate myocardial oxygen demand and contribute to cardiac stress.^5^

Atrial fibrillation/flutter was identified in 8.8% of patients, with new-onset atrial fibrillation/flutter observed in 3.4%. These arrhythmias, especially when new, were associated with poor outcomes. The pathophysiology involves systemic inflammation, catecholamine surges, hypoxemia, and structural myocardial changes, all of which promote atrial remodeling and electrical instability.^4^ Previous studies have consistently reported sinus tachycardia and atrial fibrillation as the most common rhythms observed in sepsis. Xue et al identified sinus tachycardia and atrial fibrillation as frequent findings in patients with septic cardiomyopathy.^3^ Additionally, Martin et al, Bashar et al, and L’Heureux et al emphasized that atrial fibrillation is a particularly prevalent arrhythmia in septic patients, affecting nearly 20% of cases.^2^,^4^,^9^,^22^ These arrhythmias are driven by systemic inflammation, autonomic dysregulation, and myocardial stress, all of which are hallmarks of sepsis.

The most prevalent ECG pattern abnormality was QT prolongation, found in 54% of patients. Our findings regarding the high prevalence of QT prolongation are consistent with the study by Liu et al, who reported new-onset QT prolongation in 22.9% of sepsis patients, linking it to increased mortality and tachyarrhythmias.^14^ This suggests that QT prolongation may serve as both a marker of disease severity and associated with adverse outcomes in sepsis. The pathophysiological basis of QT prolongation in sepsis includes electrolyte imbalances, systemic inflammation, and direct myocardial injury.^14^,^23^,^24^

ST elevation was observed in 10.4% of patients based on automated ECG interpretation. However, after expert review by a cardiologist, only a small fraction (1.7%) were determined to meet the diagnostic criteria for acute coronary syndrome. This discrepancy underscores the limitations of relying solely on automated ECG interpretation algorithms to interpret ECG changes in sepsis, where ST elevation may often reflect non-ischemic etiologies such as myocarditis, sepsis-induced myocardial dysfunction, or early repolarization patterns rather than true STEMI.^4^,^25^ Interestingly, patients whose ST elevation was confirmed as ACS by cardiologists demonstrated paradoxically better outcomes. This may be attributed to prompt recognition, rapid diagnostic workup, and early targeted management, such as empiric treatment for both sepsis and possible coronary syndromes, leading to timely interventions that mitigated further myocardial injury.

In contrast, abnormal T-wave findings, present in 8.9% of patients, were significantly associated with poor clinical outcomes (OR 1.34, 95% CI 1.01–1.76). These abnormalities likely reflect broader myocardial stress or repolarization disturbances, rather than being limited to ischemic T-wave inversion alone. T-wave inversion is indicative of ischemic or metabolic stress.^3^ We observed significant associations between T-wave abnormalities and poor outcomes, which is consistent with these findings.

A similar rationale could apply to other ECG patterns, such as ST depression and new-onset left bundle branch block. However, definitive conclusions cannot be drawn, as we lacked data on coronary artery disease diagnoses from coronary angiogram reports and cardiac biomarkers. Similar to our results, ST-segment abnormalities are commonly observed in sepsis, as reported by Mehta et al.^15^ However, ST elevation in sepsis does not always indicate ischemia; it may instead reflect myocarditis or sepsis-induced cardiomyopathy.^4^,^25^

## LIMITATIONS

This study benefits from a large sample size and a comprehensive analysis of ECG findings in a diverse sepsis population. However, the retrospective design and single-center setting may limit the generalizability of the findings. A primary limitation is the high exclusion rate due to missing ECG data (39.2%), which reflects real-world clinical variability in ordering practices where ECGs were not protocolized for all sepsis patients. This non-systematic data acquisition introduces a potential selection bias, as patients without clinical suspicion of cardiac issues may have been disproportionately excluded. Due to the retrospective nature of the study and reliance on a predefined dataset, we were unable to include additional clinical variables such as ED diagnoses, therapeutic interventions, or baseline functional status. These unmeasured factors could potentially confound the observed associations between ECG abnormalities and poor outcomes in sepsis.

This study did not capture data on specific clinical interventions, such as fluid resuscitation volumes, vasopressor use, or timing of antibiotics, which could independently influence both ECG changes and patient outcomes. Additionally, while chronic comorbidities were recorded, we were unable to account for acute concurrent conditions like myocardial infarction or pulmonary embolism that may have developed during the sepsis episode. Heart rate stratification could not be performed because heart rate values were derived from a single ECG recording rather than continuous monitoring, limiting our ability to categorize tachycardia severity. Future studies should evaluate heart rate trends or severity tiers as prognostic variables.

Additional limitations include the potential for SIRS to over-identify sepsis, while the. SOFA score could not be calculated because several required laboratory elements were not routinely available at ED presentation. Sensitivity analyses restricted to patients with qSOFA ≥ 2 were not performed because qSOFA functions as a mortality risk tool rather than a diagnostic criterion; relying solely on qSOFA may exclude early sepsis cases detected through SIRS-based triggers. Automated ECG interpretations also carry known inaccuracies, particularly for ST-elevation detection, and QT-interval assessment may be affected by unmeasured QT-prolonging medications. Timing variability in electrolyte testing (eg, potassium, calcium, magnesium) may also introduce misclassification of ECG-laboratory associations. Furthermore, reliance on ED-ordered ECGs introduces unavoidable selection bias, and incomplete outside-hospital ECG or medical history data may contribute to misclassification of new-onset atrial fibrillation or other abnormalities. These factors should be considered when interpreting the findings.

New-onset atrial fibrillation/flutter was identified based on the absence of prior ECGs or documented history in the EHR; however, incomplete outside-hospital ECG records may cause misclassification. This limitation prevents a more granular analysis of how these distinct clinical scenarios might impact outcomes in sepsis. Furthermore, most ECG abnormalities, including QT prolongation and T-wave abnormalities, were derived from automated ECG interpretations without physician adjudication. This may result in misclassification and should be considered when interpreting the results. Additionally, the cardiologist who reviewed ECGs flagged with ST elevation was not blinded to the study and was aware that all patients were diagnosed with sepsis. This lack of blinding may have influenced interpretation, particularly in borderline cases, and represents a potential source of observer bias.

The overall 18% poor-outcome rate observed in our cohort was driven primarily by prolonged hospital LOS (> 14 days) rather than ICU admission or in-hospital mortality. This outcome structure may limit interpretability and should be considered when comparing with sepsis cohorts that use mortality-based endpoints. Additionally, LOS can be affected by non-clinical system factors such as bed availability and discharge delays. This may introduce variability that weakens its role as a stand-alone measure of poor outcomes. Additionally, most of the composite events were driven by prolonged hospitalization, which may overestimate clinical deterioration.

Although septic shock cases were included in the cohort, subgroup stratification was not performed because the study aimed to evaluate ECG abnormalities across the full sepsis spectrum rather than outcomes specific to septic shock.

Multivariable regression was not performed because key confounders such as illness severity, intervention timing, and QT-prolonging medications were incompletely captured, and adjusted estimates under these conditions could be unreliable. Therefore, there is a need for prospective multicenter studies to validate these findings and to further explore the mechanisms linking ECG abnormalities to sepsis outcomes.

## CONCLUSION

This study highlights the prevalence and prognostic significance of ECG abnormalities in sepsis patients. Atrial fibrillation/flutter and QT prolongation were associated with poor outcomes, while sinus rhythm was more frequently observed among patients with favorable outcomes. These findings show that ECG abnormalities are associated with adverse outcomes and may help identify higher risk sepsis patients early in their ED stay.

## Figures and Tables

**Figure:**
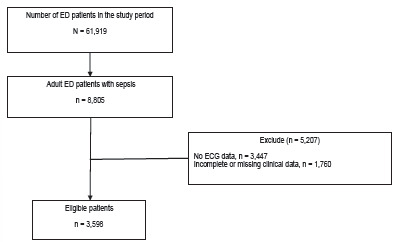
Flow diagram of patient selection in a retrospective study of electrocardiographic findings in ED patients with sepsis. *ED*, emergency department; *ECG*, electrocardiogram.

**Table 1:** Baseline demographic, clinical, and laboratory characteristics of adult sepsis patients in a retrospective study conducted in the ED, stratified by clinical outcomes.

Characteristic	Overall, N = 3,598	Better outcome, n = 2,949	Poor outcome, n = 649
Male, n (%)	1,984 (55.1%)	1,595 (54.1%)	389 (59.9%)
Age (years)			
18–39, n (%)	432 (12.0%)	359 (12.2%)	73 (11.2%)
40–59, n (%)	857 (23.8%)	701 (23.8%)	156 (24.0%)
≥60, n (%)	2,309 (64.2%)	1,889 (64.0%)	420 (64.7%)
Mean age, mean (SD)	63 (18)	63 (18)	63 (17)
Vital signs			
Body temperature (°C), mean (SD)	37.6 (1.1)	37.6 (1.1)	37.4 (1.1)
Heart rate (bpm), mean (SD)	105 (20)	105 (20)	106 (21)
Respiratory rate (bpm), mean (SD)	26 (7)	26 (7)	27 (7)
Systolic blood pressure (mm Hg), mean (SD)	134 (29)	135 (29)	129 (29)
Diastolic blood pressure (mm Hg), mean (SD)	78 (18)	78 (17)	77 (19)
Peripheral oxygen saturation (%), mean (SD)	95.3 (6.4)	95.6 (6.0)	94.2 (7.9)
Underlying disease			
Myocardial infarction, n (%)	175 (4.9%)	138 (4.7%)	37 (5.7%)
Heart failure, n (%)	437 (12.1%)	336 (11.4%)	101 (15.6%)
Atrial fibrillation/flutter, n (%)	529 (14.7%)	373 (12.6%)	156 (24.0%)
Stroke/TIA, n (%)	481 (13.4%)	391 (13.3%)	90 (13.9%)
Pulmonary embolism, n (%)	162 (4.5%)	118 (4.0%)	44 (6.8%)
COPD, n (%)	302 (8.4%)	232 (7.9%)	70 (10.8%)
Liver disease, n (%)	257 (7.1%)	193 (6.5%)	64 (9.9%)
Chronic kidney disease, n (%)	708 (19.7%)	570 (19.3%)	138 (21.3%)
Diabetes mellitus, n (%)	1,296 (36.0%)	1,053 (35.7%)	243 (37.4%)
Hypertension, n (%)	1,991 (55.3%)	1,615 (54.8%)	376 (57.9%)
Dyslipidemia, n (%)	470 (13%)	401 (14%)	69 (11%)
Any cancers, n (%)	32 (0.9%)	26 (0.9%)	6 (0.9%)
SIRS score ≥ 2, n (%)	3,472 (96.5%)	2,849 (96.6%)	623 (96.0%)
qSOFA score ≥ 2, n (%)	737 (20.5%)	553 (18.8%)	184 (28.3%)
Laboratory results			
White blood cell count (cell/mm^3^), mean (SD)	12,677 (9,309)	12,444 (9,162)	13,738 (9,889)
Creatinine (mg/dL), mean (SD)	1.47 (1.65)	1.46 (1.65)	1.55 (1.65)
Potassium (mEq/L), mean (SD)	4.07 (0.71)	4.06 (0.70)	4.12 (0.77)
Calcium (mg/dL), mean (SD)	8.6 (0.9)	8.7 (0.8)	8.5 (1.1)
Magnesium (mg/dL), mean (SD)	1.9 (0.4)	1.9 (0.3)	1.9 (0.4)
Bicarbonate (mEq/L), mean (SD)	20.9 (4.2)	20.9 (4.0)	20.6 (5.0)
Serum lactate (mmol/L), mean (SD)	2.6 (2.3)	2.7 (2.2)	3.1 (2.8)

*COPD*, chronic obstructive pulmonary disease; *ED*, emergency department; *qSOFA*, Quick Sequential Organ Failure Assessment; *SIRS*, Systemic Inflammatory Response Syndrome; *TIA*, transient ischemic attack.

**Table 2:** Electrocardiographic rhythm findings in adult sepsis patients presenting to the emergency department.

	Overall, N = 3,598	Better outcome, n = 2,949	Poor outcome, n = 649	Odds ratio (95%)	P-value
Rhythms					
Sinus rhythm	1,501 (41.7%)	224 (34.5%)	224 (34.5%)	0.69 (0.58–0.82)	<0.001
Sinus tachycardia	1,402 (39.0%)	272 (41.9%)	272 (41.9%)	1.16 (0.98–1.38)	.09
Sinus bradycardia	41 (1.1%)	7 (1.1%)	7 (1.1%)	0.93 (0.38–1.99)	.87
Sinus arrhythmia	8 (0.2%)	2 (0.3%)	2 (0.3%)	1.52 (0.22–6.60)	.61
Atrial fibrillation/Atrial flutter	318 (8.8%)	97 (14.9%)	97 (14.9%)	2.19 (1.77–2.69)	<0.001
New Atrial fibrillation/Atrial flutter	121 (3.4%)	39 (6.0%)	39 (6.0%)	2.24 (1.50–3.28)	<0.001
Atrial tachycardia	44 (1.2%)	9 (1.4%)	9 (1.4%)	1.17 (0.53–2.34)	.68
Supraventricular tachycardia	16 (0.4%)	4 (0.6%)	4 (0.6%)	1.52 (0.42–4.37)	.47
Ventricular arrhythmia	18 (0.5%)	2 (0.3%)	2 (0.3%)	0.57 (0.09–2.00)	.45
Atrioventricular block					
1st degree	237 (6.6%)	35 (5.4%)	35 (5.4%)	0.78 (0.53–1.11)	.18
2nd degree	3 (<0.1%)	0 (0.0%)	0 (0.0%)	NA (NA–1.92×10^12^)	.97
3rd degree	1 (<0.1%)	0 (0.0%)	0 (0.0%)	NA (NA–1.17×10^13^)	.96
Frequent atrial premature beat	145 (4.0%)	30 (4.6%)	30 (4.6%)	1.19 (0.78–1.78)	.40
Frequent ventricular premature beat	233 (6.5%)	53 (8.2%)	53 (8.2%)	1.37 (0.99–1.87)	.05
Frequent junctional rhythm	5 (0.1%)	1 (0.2%)	1 (0.2%)	1.14 (0.06–7.70)	.91

**Table 3:** Abnormal electrocardiographic patterns observed in sepsis patients presenting to the emergency department.

Patterns	Overall, N = 3,598	Better outcome, n = 2,949	Poor outcome, n = 649	Odds ratio (95% CI)	P-value
PR prolonged	222 (6.2%)	190 (6.4%)	32 (4.9%)	0.75 (0.50–1.09)	.15
QRS prolonged	256 (7.1%)	204 (6.9%)	52 (8.0%)	1.17 (0.85–1.60)	.33
QT prolonged	1,960 (54.4%)	1,560 (52.9%)	400 (61.6%)	1.43 (1.20–1.70)	<0.001
New pathological Q wave	20 (0.6%)	14 (0.5%)	6 (0.9%)	1.96 (0.69–4.90)	.17
ST-T changes					
ST elevation	374 (10.4%)	320 (10.9%)	54 (8.3%)	0.75 (0.55–1.00)	.06
ST depression	96 (2.7%)	80 (2.7%)	16 (2.5%)	0.91 (0.51–1.52)	.72
Abnormal T wave	319 (8.9%)	248 (8.4%)	71 (10.9%)	1.34 (1.01–1.76)	.04
ACS diagnosis by cardiologist	62 (1.7%)	53 (1.8%)	9 (1.4%)	0.77 (0.35–1.49)	.47
Early repolarization	306 (8.5%)	255 (8.6%)	51 (7.9%)	0.90 (0.65–1.22)	.51
Left bundle branch block	45 (1.3%)	38 (1.3%)	7 (1.1%)	0.84 (0.34–1.77)	.66
New left bundle branch block	22 (0.6%)	18 (0.6%)	4 (0.6%)	1.01 (0.29–2.72)	.99
Right bundle branch block	111 (3.1%)	83 (2.8%)	28 (4.3%)	1.56 (0.99–2.38)	.05
Non-specific intraventricular conduction delay	76 (2.1%)	62 (2.1%)	14 (2.2%)	1.03 (0.55–1.79)	.93

*ACS*, acute coronary syndrome.

## References

[1] Singer M, Deutschman CS, Seymour CW (2016). The Third International Consensus Definitions for Sepsis and Septic Shock (Sepsis-3). JAMA.

[2] Martin L, Derwall M, Al Zoubi S (2019). The septic heart: current understanding of molecular mechanisms and clinical implications. Chest.

[3] Xue W, Pang J, Liu J (2022). Septic cardiomyopathy: characteristics, evaluation, and mechanism. Emerg Crit Care Med.

[4] L’Heureux M, Sternberg M, Brath L (2020). Sepsis-induced cardiomyopathy: a comprehensive review. Curr Cardiol Rep.

[5] Kakihana Y, Ito T, Nakahara M (2016). Sepsis-induced myocardial dysfunction: pathophysiology and management. J Intensive Care.

[6] Beesley SJ, Weber G, Sarge T (2018). Septic cardiomyopathy. Crit Care Med.

[7] Lin YM, Lee MC, Toh HS (2022). Association of sepsis-induced cardiomyopathy and mortality: a systematic review and meta-analysis. Ann Intensive Care.

[8] van Wijk RJ, Quinten VM, van Rossum MC (2023). Predicting deterioration of patients with early sepsis at the emergency department using continuous heart rate variability analysis: a model-based approach. Scand J Trauma Resusc Emerg Med.

[9] Bashar SK, Ding EY, Walkey AJ (2021). Atrial fibrillation prediction from critically ill sepsis patients. Biosensors.

[10] Rich MM, McGarvey ML, Teener JW (2002). ECG changes during septic shock. Cardiology.

[11] Ishmael L, Zalocha J (2020). ST-Elevation myocardial infarction in the presence of septic shock. Case Rep Crit Care.

[12] Sandoval Y, Jaffe AS (2019). Type 2 Myocardial infarction: JACC review topic of the week. J Amer Coll Cardiol.

[13] Kwon JM, Lee YR, Jung MS (2021). Deep-learning model for screening sepsis using electrocardiography. Scand J Trauma Resusc Emerg Med.

[14] Liu W, Shao R, Zhang S (2024). Characteristics, predictors and outcomes of new-onset QT prolongation in sepsis: a multicenter retrospective study. Crit Care.

[15] Mehta S, Granton J, Lapinsky SE (2011). Agreement in electrocardiogram interpretation in patients with septic shock. Crit Care Med.

[16] Bone RC, Balk RA, Cerra FB (1992). Definitions for sepsis and organ failure and guidelines for the use of innovative therapies in sepsis. Chest.

[17] Rao SV, O’Donoghue ML, Ruel M (2025). 2025 ACC/AHA/ACEP/NAEMSP/SCAI guideline for the management of patients with acute coronary syndromes: a report of the American College of Cardiology/American Heart Association Joint Committee on Clinical Practice Guidelines. Circulation.

[18] Worster A, Bledsoe RD, Cleve P (2005). Reassessing the methods of medical record review studies in emergency medicine research. Ann Emerg Med.

[19] Rodrigues AR, Oliveira A, Vieira T (2024). A prolonged intensive care unit stay defines a worse long-term prognosis – Insights from the Critically Ill Mortality by Age (Cimba) study. Aust Crit Care.

[20] Cheung Y, Ko S, Wong OF (2021). Clinical experience in management of bloodstream infection in emergency medical ward: A preliminary report. Hong Kong J Emerg Med.

[21] Li C, Wong O, Ko S (2018). A preliminary report of clinical experience in managing patients with sepsis and septic shock in emergency medicine ward. Hong Kong J Emerg Med.

[22] Klein Klouwenberg PMC, Frencken JF, Kuipers S (2017). Incidence, predictors, and outcomes of new-onset atrial fibrillation in critically ill patients with sepsis. a cohort study. Am J Respir Crit Care Med.

[23] Kim ED, Watt J, Tereshchenko LG (2019). Associations of serum and dialysate electrolytes with QT interval and prolongation in incident hemodialysis: the Predictors of Arrhythmic and Cardiovascular Risk in End-Stage Renal Disease (PACE) study. BMC Nephrol.

[24] Lazzerini PE, Laghi-Pasini F, Bertolozzi I (2017). Systemic inflammation as a novel QT-prolonging risk factor in patients with torsades de pointes. Heart.

[25] Sato R, Nasu M (2013). A review of sepsis-induced cardiomyopathy. J Intensive Care.

